# The Status of Children’s Dental Health in Rural Appalachian West Virginia

**DOI:** 10.13023/jah.0703.09

**Published:** 2025-09-01

**Authors:** R. Constance Wiener, Gina Graziani

**Affiliations:** West Virginia University; West Virginia University

**Keywords:** Appalachia, Metro, National Survey of Children’s Health, Oral health, rural, West Virginia

## Abstract

**Introduction:**

Non-metro/rural Appalachian West Virginia (WV) residents have been stigmatized for poor oral health (OH), despite many advances.

**Purpose:**

The aims of this study were to examine current OH in children in WV, as compared to children in the greater United States (U.S.), and secondarily in subgroup non-metro/rural comparisons.

**Methods:**

This observational study involved the U.S. National Survey of Children’s Health 2022–2023 data from parents/guardians who were asked about their child’s previous 12-month OH. Rao-Scott Chi-square and logistic regression analyses were used.

**Results:**

Nationally, the mean age was 9.7 years; for WV children, it was 9.9 years. Nationally, 12.4% of children lived in non-metro/rural areas; in WV, 36.5% of children lived in non-metro/rural areas. Children from WV were similar or had slightly more positive outcomes of being more likely to see a dentist, have a dental prophylaxis, have a professional fluoride treatment, have sealant placement, and to have both preventive dental and medical care, as compared to children in the rest of the nation. Among non-metro/rural children, WV children were more likely to see a dentist and have ≥1 preventive dental visit(s), dental prophylaxis, oral hygiene instructions, fluoride, sealant(s), and to have both preventive dental and medical care.

**Implications:**

Children living in WV have similar or slightly better OH than children living in the U.S. overall. Similarly, children living in non-metro/rural WV have similar or slightly better OH than children living in non-metro/rural U.S. These positive results are often obscured by the previous health history in rural WV. Overall, there remains a need to continue to improve OH, particularly in improving the number of children who have preventive dental care.

## INTRODUCTION

Studying oral health (OH) in rural states, such as West Virginia (WV), remains important, as there are many public health challenges identified in rural areas: lack of insurance, poverty,^1^ education,^2^ and provider shortages. For example, WV has 49 dentists per 100,000 residents,^3^ while the greater United States (U.S.) has 60.84 per 100,000, as of 2021.^4^ There is also the issue of rural time-distance barriers for OH visits in WV.^5^ For rural Medicaid-managed primary care, the mean travel-time to providers is 44.7 minutes, compared to 28.9 minutes for urban residents.^5^ Nevertheless, OH in some U.S. rural regions has had recent successes. Success in WV is often overlooked due to persistent prejudices about rurality, lack of reliable and recent data, and the use of reporting measures which hide existent progress.^6^ Descriptors of preventive dental service utilization and self-assessment of OH provide detailed appraisals of incident OH status, particularly for children. There is limited current data about OH progress of WV children as it compares with the rest of the nation with such descriptors. Therefore, the aims of this study were to examine (1) current OH conditions of children in WV v children overall in the U.S. (excluding WV); and (2) current OH conditions of non-metro/rural children in WV versus non-metro/rural children in the U.S. (excluding WV).

## METHODS

Data for this cross-sectional study were of non-institutionalized U.S. children from years 2022 (*n*=54,103) and 2023 (*n*=55,162) of the U.S. National Survey of Children’s Health (NSCH)^7^ designed by the Maternal and Child Health Bureau and Services Administration; Census Bureau; National Center for Health Statistics (CDC); Child and Adolescent Health Measurement Initiative; and an expert panel.^7^.^8^ NSCH questions were presented to parents/guardians who responded about demographics, social factors, healthcare availability, and child/family health, including the OH status of their child over the previous 12 months. They were asked about having seen a dentist and/or other healthcare provider, if there were any OH problems (e.g. toothaches, bleeding gingiva, dental caries), examinations, preventive visits, prophylaxis, oral hygiene instructions, radiographs, professional fluoride, dental sealants, and having both preventive dental and preventive medical care (Table 1). The NSCH provides sample weights for the data to be representative of the U.S. population at large.^7^

For this current study, dental data for children (ages 1–17 years) from the national and WV metro and non-metro/rural designations were used.

**Fig 1:**
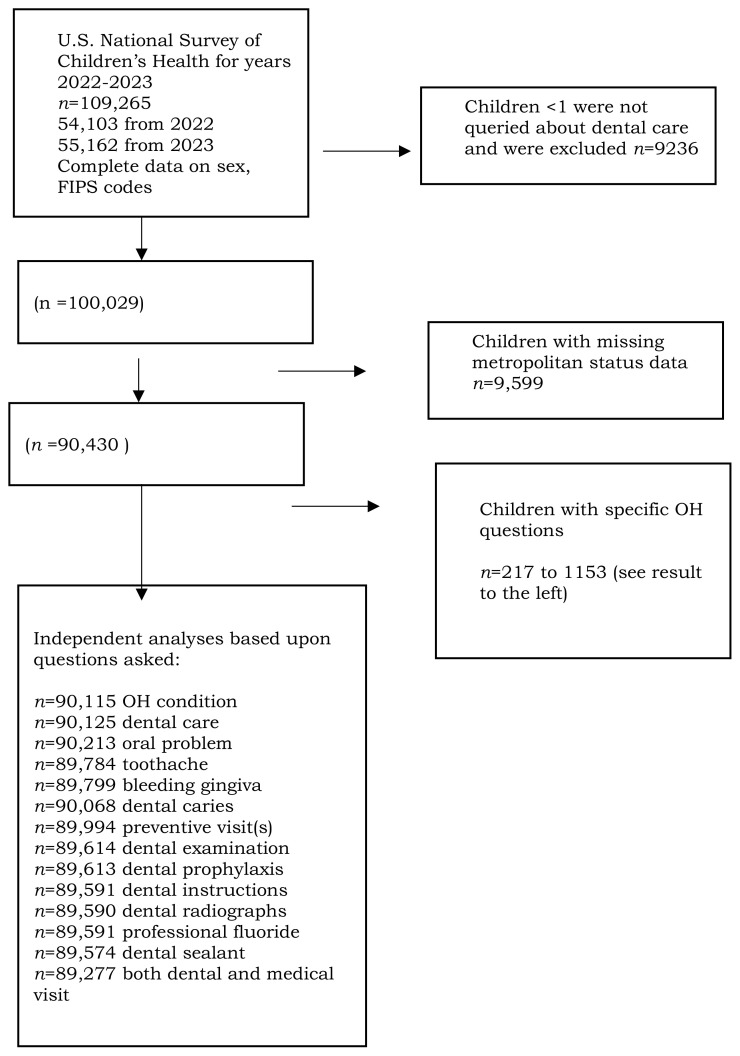
Flow chart of inclusion/exclusion criteria for OH

The U.S. Census Bureau defines rural as the population, housing, and territory not included in an urban area.^9^ The NSCH uses the variable METRO_YN (yes, no) for this designation; this variable was used in this study. The eligible inclusion criteria were complete data on sex, age, federal information processing standards (identifying states and associated areas), and a response to having had a dental visit within the previous year (Fig. 1). Race, family structure, poverty level, language spoken at home, and insurance status were included in the logistic regression. *A priori*, *p* <.05 was determined as the significance level. National data were extracted using SAS® version 9.4. Data weights and strata provided in the data set were used in the analyses. Rao-Scott Chi-Square test and Phi coefficient calculation were conducted on the above-mentioned OH outcomes. Adjusted logistic regression analyses were conducted for each question concerning OH controlling for sex, age, family structure, poverty level, language spoken at home, and insurance status.

### Ethics Approval

This study was reviewed by the West Virginia University Institutional Review Board and was determined to be a non-human research study (Certificate 1148).

## RESULTS

### Comparison of WV and Nationwide (Excluding WV)

Nationally, the mean age for this sample was 9.7 years (standard error of mean = 0.03; *n* = 88,919) and for WV children, it was 9.9 years (standard error of mean = 0.15; *n* = 1,511).

WV children had either similar or more positive OH than national children (excluding WV) (Table 1). WV children were more likely than national children (excluding WV) to see a dentist (87.1% [95%CI:84.8%–89.5%] and 83.8% [95%CI:83.3–84.4], respectively), have a dental prophylaxis (80.8% [95CI:78.1–83.6] and 75.7% [95%CI:75.1–76.3], respectively), have a professional fluoride treatment (53.7% [95%CI:50.3%, 57.1%] and 47.7% [95%CI:47.0–48.3%], respectively), have a dental sealant (19.7% [95%CI:16.9–22.4], respectively), and to have both preventive dental and medical care within the previous 12 months (72.2% [95%CI: 69.0–75.4] and 66.1 [95%CI: 65.5–66.8], respectively).

For both WV and the nation, approximately three-fourths of children were reported to have teeth that were in good/very good/excellent condition (94.8% [95%CI:93.0–96.6] and 94.1% [95%CI: 93.7–94.4], respectively), and to have had a dental examination within the past 12 months (78.2% [95%CI:75.3–81.1], and 71.7%[95%CI: 75.1–76.3], respectively). Most had no OH problems (84.2% [95%CI:81.6–86.8] and 85.4% [95%CI:84.9–85.9], respectively). There were 84.4% [95%CI: 81.9–87.0] who had at least one preventive dental visit in WV and 81.8% [95%CI:81.3–82.4] in the nation.

Adjusted odds ratios comparing WV children to national children on OH, controlled for sex, race, metropolitan status, age, family structure, poverty level, language spoken at home, and insurance status are also provided in Table 1.

### Comparison of Non-Metro/Rural WV and Non-Metro/Rural U.S. (Excluding WV)

Nationwide, 12.4% of respondents were non-metro/rural (*n* = 14,480), whereas 36.5% of WV respondents were non-metro/rural (*n* = 490). WV non-metro/rural children had either similar or more positive OH outcomes than national non-metro/rural children (excluding WV) (Table 2). WV non-metro/rural children were more likely than national non-metro/rural children to see a dentist (85.6% [95%CI: 81.2–90] and 79.8% [95%CI: 78.5–81.1], respectively), have ≥1 preventive dental visit(s) (85.2 [95%CI: 80.6–89.7] and 78.0% [95%CI:76.7–79.4], respectively), dental prophylaxis (80.9% [95%CI:76.1–85.7] and 71.7 [95%CI:70.3–73.1], respectively), oral hygiene instructions (47.9 [95%CI:42.1–53.7] and 40.9% [95%CI: 39.4–42.5], respectively), professional fluoride (54.8% [95%CI:49.0–60.6] and 47.2 [95%CI:45.7–49.8], respectively), dental sealant(s) (23.2% [95%CI:18.3–28.0] and 16.6% [95%CI: 15.4–17.7], respectively), and to have both preventive dental and medical care within the previous 12 months (72.8% [95%CI:67.3–78.3] and 60.6% [95%CI: 59.0–62.2], respectively).

For both non-metro/rural WV children and the non-metro/rural national children, approximately three-fourths of children were reported to have teeth that were in good/very good/excellent condition (91.2% [95%CI: 87.1–95.3] and 92.1% [95%CI: 91.1–93.1], respectively). There were 76.5% [95%CI:71.4–81.7] of WV non-metro/rural children and 71.2% [95%CI:69.8–72.7] of national non-metro/rural children who had a dental examination within the past 12 months. Most had no OH problems (79.3%[95%CI:74.3–84.3] for WV and 82.5% [95%CI:81.2–83.7] for the nation.

Adjusted odds ratios comparing non-metro/rural WV children to non-metro/rural national children on OH outcomes, controlled for sex, race, metropolitan status, age, family structure, poverty level, language spoken at home, and insurance status are also provided in Table 2.

## DISCUSSION

The results of overall and sub-group (non-metro/rural) comparisons of WV and national children on OH outcomes were that WV children had similar or slightly better OH outcomes than children nationwide. For example, WV children were more likely to see a dentist, have a dental prophylaxis, receive a professional application of fluoride, have dental sealants, and receive both preventive dental and medical visits within the past year than the national children in both overall and non-metro/rural analyses.

There are limited studies with which to compare these results. In a review including OH measures and outcomes in WV, there was a finding of greater use of sealants in WV than nationwide.^10^ That result supports the findings in this study. However, the cited report differs from this current study in that it was limited to WV children in third grade. This study used data of children ages 1–17 years; additionally, the report involved ever having a sealant.^10^ The findings of this current study were of having a sealant within the previous year. In the report 62.2% of WV third grade students had dental sealants on permanent molars compared to 41.5% nationally.^10^ Within the same report, 19% of third grade students had untreated dental caries. In this current study, past 12-month dental caries in WV was 13.8%. Other recent comparator studies are limited, emerging, or were unavailable.

## IMPLICATIONS

Misconceptions about OH in WV and in rural areas in the greater U.S. persist, despite progress. Educational opportunities, internet and cell phone connectivity, social media, and travel are ongoing improvements in addressing much of the isolation and lack of access previously experienced.^10^ Utilization of teledentistry has been reported to improve access, treatment, and OH in general.^11^ Efforts exist to increase teledentistry and technologies in preventive (at-home) dental care in WV.^10^, ^12^ It is hoped that with greater access to knowledge, improved delivery of care, newer materials, and technology, and greater access with programs providing travel services for care, all children will have better outcomes. However, negative perceptions about OH in WV need to be considered as well so that stigmatizing narratives about WV and the Appalachian culture can be reshaped. Many initiatives and robust investments, such as those with the Appalachian Regional Commission, are doing so. The data in this study support their impact.

### Strengths and Limitations

This study is strengthened by the availability of a nationally conducted survey with two years of data concerning OH. Data weights and strata provided in the data set were used in the analyses, making the results representative of the nation and WV. The study is limited by potential responders’ recall bias and social desirability bias. Though not a limitation, the study design does preclude establishing a causal effect to the results.

## CONCLUSION

There remains a need across the U.S. to continue to improve OH, particularly in improving the number of children who have preventive dental care.

**SUMMARY BOX**

**What is already known about this topic?**

Misconceptions about oral health in WV (and non-metro/rural WV in particular) persist, despite progress. Generally, the data available are based upon adult outcomes.

**What is added by this report?**

This report demonstrates WV child oral health outcomes are similar to or better than national levels.

**What are the implications for future research?**

Although WV oral health outcomes for children are similar to the nation, there remains a need to reach the nearly 13% of WV children and 16% of U.S. children who have not seen a dentist or other oral healthcare provider within the past 12 months.

## Figures and Tables

**Table 1:** Comparison of National and WV Dental Services Received by Children Ages 1–17 years, National Survey of Children’s Health, 2022–2023, *n* = 90,430

	Number Nation-wide (excludes WV)	Generalized Nationwide Population Estimate of Children Represented	Nation-wide % (Confidence Interval)	Number WV	Generalized WV Population Estimate of Children Represented	WV % (Confidence Interval)	*Rao-Scott Chi Square p*-value (Phi)	*Adjusted Odds Ratio Comparing WV to the Nation (95% Confidence Interval)*	*p-*Value
**The number of children whose parents reported excellent/very good condition of teeth (“Excellent/very good/good” is event modeled)**	84,725	58037875	94.1 (93.7–94.4)	1,446	303,754	94.8 (93.0,96.6)	0.4497 (0.0025)	1.36 (0.94–1.96)	0.1064
**Did not see a dentist and/or other oral healthcare provider during past 12 months (“Yes, visit” is the event modeled)**	13,781	9,980,743	16.2 (15.6–16.7)	211	41,331	12.9 (10.5, 15.2))	0.0132 (0.0132)	1.49 (1.19–1.86)	0.0004
**Had no oral health problems during past 12 months (“No problem” is the event modeled)**	77,782	52,737,054	85.4 (84.9–85.9)	1,310	271,435	84.2 (81.6–86.8)	0.3581 (0.0031)	1.01 (0.83–1,25)	0.8678
**Had no toothaches during past 12 months (“No toothache” is the event modeled)**	85,522	59,004,954	96.1 (95.8–96.3)	1,441	303,634	94.7 (93.0–96.5)	0.0891 (0.0057)	0.87 (0.60–1.24)	0.4339
**Had no bleeding gingiva during past 12 months (“No gingival bleeding” is the event modeled)**	86,925	60,246,824	98.1 (97.9–98.3)	1484	316,106	98.5 (97.6–99.4)	0.5189 (0.0024)	1.50 (0.78–2.89)	0.2270
**Had no dental caries during past 12 months (“No caries” is the event modeled)**	79,084	53,779,462	87.2 (86.8–87.7)	1,330	277,396	86.2 (83.7–88.7)	0.4116 (0.027)	1.03 (0.83–1.27)	0.8148
**Had 1 preventive dental visit during past 12 months (“Had preventive” is the event modeled)**	73,369	50,361,685	81.8 (81.3–82.4)	1,261	217,779	84.4 (81.9–87.0)	0.0681 (0.0061)	1.35 (1.10–1.66)	0.0045
**Had a dental examination during past 12 months (“Had examination” Is the event modeled)**	68,947	46,370,818	75.7 (75.1–76.3)	1,168	250,475	78.2 (75.3, 81.1)	0.1136 (0.0053)	1.30 (1.08–1.56)	0.0047
**Had a dental prophylaxis during past 12 months (“Had prophylaxis” is the event modeled)**	67,404	46,377,082	75.7 (75.1, 76.3)	1,176	258,994	80.8 (78.1 83.6)	0.0010 (0.0110)	1.53 (1.26–1.84)	<.0001
**Had oral hygiene instructions during past 12 months (“Had Instructions” is the event modeled)**	42,558	27,254,609	44.5 (43.9–45.2	687	149,425	46.6 (43.2, 50.0)	0.2300 (0.0040)	1.20 (1.04–1.49)	0.0152
**Had radiographs during past 12 months (“Had radiographs” is the event modeled)**	44,720	30,447,568	49.7 (49.1–50.4)	727	161,656	50.4 (47.0, 53.8)	0.6815 (0.0014)	1.11 (0.96–1.29)	0.1761
**Received professional fluoride treatment during past 12 months (“Had fluoride” is the event modeled)**	45,681	29,200,803	47.7 (47.0–48.3)	784	172,154	53.7 (50.3, 57.1)	0.0007 (0.0113)	1.34 (1.15–1.55)	0.0001
**Received a dental sealant during the past 12 months (“Had sealant” is the event modeled)**	14,033	9,801,796	16.0 (15.5–16.5)	256	63,020	19.7 (16.9, 22.4)	0.0058 (0.0092)	1.29 (1.07–1.54)	0.0071
**Received both preventive dental and medical care during the past 12 months (“Had both dental and medical care” is the event modeled)**	61,095	40,329,197	66.1 (65.5–66.8)	1108	227,684	72.2 (69.0, 75.4)	0.0005 (0.0116)	1.49 (1.26–1.77)	<.0001

Note: Adjusted logistic regression controlled for sex, race, rural/urban status, age, family structure, poverty level, language spoken at home, and insurance status.

**Table 2:** Comparison of Non-metro/rural National and Non-metro/rural WV dental services received by children ages 1–17 years, National Survey of Children’s Health, 2022–2023, *n* = 15,967

	Number Nationwide (excluding WV) non-metro/rural	Generalized Nationwide Population estimate of Non-metro/rural children represented	Nationwide % (Confidence Interval)	Number WV Non-metro/rural	Generalized WV population estimate of non-metro/rural children represented	WV % (Confidence Interval)	Rao-Scott Chi Square *p*-value (Phi)	*Adjusted Odds Ratio comparing WV to the nation (95% Confidence Interval)*	*p-*value
**The number of children whose parents reported excellent/very good condition of teeth (“Excellent/very good/good” is the event modeled)**	14,480	7,012,121	92.1 (91.1–93.1)	490	105,821	91.2 (87.1, 95.3)	0.6513 (0.0036)	0.97 (0.56–1.66)	0.9023
**Did not see a dentist and/or other oral healthcare provider during past 12 months (“Yes, visit” is the event modeled)**	2,847	1,536,211	20.2 (18.9–21.5)	72	16,785	14.4 (10.0, 18.8)	0.0281 (0.0174)	1.56 (1.06–2.27)	0.0228
**Had no oral health problems during past 12 months (“No problems” is the event modeled)**	13,055	6,293,470	82.5 (81.2–83.7)	438	93,343	79.3 (74.3–84.3)	0.2056 (0.0100)	0.90 (0.66–1.25)	0.5411
**Had no toothaches during past 12 months (“No toothache” is the event modeled)**	14,718	7,180,783	94.4 (93.5–95.3)	498	110,112	93.9 (90.6–97.2)	0.0927 (0.0027)	1.08 (0.58–2.00)	0.8169
**Had no bleeding gingiva during past 12 months (“No bleeding gingiva” is the event modeled)**	15,052	7,419,798	97.6 (97.2–98.1)	512	115,336	98.4 (97.1, 99.6)	0.3542 (0.0059)	1.75 (0.77–4.00)	0.1823
**Had no dental caries during past 12 months (“No caries” is the event modeled)**	13,312	6,440,781	84.5 (83.3–85.7)	443	94,848	80.7 (75.8–85.6)	0.1101 (0.0128)	0.85 (0.61–1.18)	0.3232
**Had 1 preventive dental visit during past 12 months (“Had preventive” is event modeled)**	12,261	5,924,343	78.0 (76.7–79.4)	445	99,770	85.2 (80.6–89.7)	0.0398 (0.0211)	1.71 (1.17–2.50)	0.0057
**Had a dental examination during past 12 months**	11,384	5,378,425	71.2 (69.8–72.7)	401	89,572	76.5 (71.4, 81.7)	0.0669 (0.0144)	1.39 (1.02–1.90)	0.0364
**Had a dental prophylaxis during past 12 months (“Had prophylaxis” is the event modeled)**	11,136	5,413,385	71.7 (70.3–73.1)	415	94,641	80.9 (76.1, 85.7)	0.0015 (0.0250)	1.77 (1.27–2.47)	0.0008
**Had oral hygiene instructions during past 12 months (“Had instructions” is the event modeled)**	6,805	3, 091,019	40.9 (39.4–42.5)	239	56,035	47.9 (42.1, 53.7)	0.0219 (0.0173)	1.38 (1.08–1.78)	0.0104
**Had radiographs during past 12 months (“Had radiographs” is the event modeled)**	7,220	3,502,085	46.4 (44.8–47.9)	249	60,538	51.7 (46.0, 57.5)	0.0784 (0.0132)	1.28 (1.00–1.65)	0.0516
**Received professional fluoride treatment during past 12 months (“Had fluoride” is the event modeled)**	7.645	3,566,340	47.2 (45.7–48.8)	274	64,130	54.8 (49.0, 60.6)	0.0136 (0.0186)	1.40 (1.09–1.80)	0.0087
**Received a dental sealant during the past 12 months (“Had sealant” is the event modeled)**	2,559	1,250,985	16.6 (15.4–17.7)	108	27,126	23.2 (18.3, 28.0)	0.0037 (0.0217)	1.53 (1.14–2.06)	0.0048
**Received both preventive dental and medical care during the past 12 months (“Had both dental and medical care” is the event modeled)**	9,778	4,567,251	60.6 (59.0–62.2)	388	83,300	72.8 (67.3, 78.3)	0.0001 (0.0303)	1.83 (1.36–2.46)	<.0001

NOTE: Adjusted logistic regression controlled for sex, race, rural/urban status, age, family structure, poverty level, language spoken at home, and insurance status.
